# Residual radiological opacities correlate with disease outcomes in ICU-treated COVID-19

**DOI:** 10.3389/fmed.2024.1263511

**Published:** 2024-04-03

**Authors:** Mikael Björnson, Ann Mari Svensson, Cecilia He, Magnus Sköld, Sven Nyrén, Malin Nygren-Bonnier, Judith Bruchfeld, Michael Runold, Francesca Campoccia Jalde, Anna Kistner

**Affiliations:** ^1^Department of Infectious Diseases, Karolinska University Hospital, Stockholm, Sweden; ^2^Department of Medicine Solna, Division of Infection Diseases, Karolinska Institutet, Stockholm, Sweden; ^3^Department of Radiology, Karolinska University Hospital, Stockholm, Sweden; ^4^Department of Molecular Medicine and Surgery, Karolinska Institutet, Stockholm, Sweden; ^5^Department of Respiratory Medicine and Allergy, Karolinska University Hospital, Stockholm, Sweden; ^6^Respiratory Medicine Unit, Department of Medicine Solna and Center for Molecular Medicine, Karolinska Institutet, Stockholm, Sweden; ^7^Department of Neurobiology, Care Sciences and Society, Division of Physiotherapy, Karolinska Institutet, Huddinge, Sweden; ^8^Women’s Health and Allied Health Professionals Theme, Medical Unit Allied Health Professionals, Karolinska University Hospital, Stockholm, Sweden; ^9^Department of Cardiothoracic Surgery and Intensive Care, Karolinska University Hospital, Stockholm, Sweden; ^10^Department of Physiology and Pharmacology, Karolinska Institutet, Stockholm, Sweden; ^11^Medical Radiation Physics and Nuclear Medicine, Karolinska University Hospital, Stockholm, Sweden

**Keywords:** COVID-19, computed tomography, spirometry, exercise test, pulmonary diffusing capacity, residual radiological opacities

## Abstract

**Background:**

Few studies consider both radiological and functional outcomes in COVID-19 survivors treated in the intensive care unit (ICU). We investigated clinical findings and pulmonary abnormalities on chest computed tomography (CT) and compared outcomes of severe versus mild-moderate acute respiratory distress syndrome (ARDS) on long-term follow-up.

**Methods:**

This longitudinal cohort study included 118 COVID-19 patients (median age, 58 years; 79% men). Thoracic CT scans were performed 4, 10, and 22 months after hospital discharge. Two independent blinded radiologists analyzed the 10 months scans and scored the radiology findings semi-quantitatively, as no/minor versus widespread opacities [low-radiology opacity grade (ROG) versus high-ROG]. ARDS severity was based on the PaO2/FiO2 ratio. The 6 min walk test (6MWT) was performed after 3 and 9 months, and lung diffusion capacity for carbon monoxide (DLCO) and lung volume measurement after 9 and 15 months. Dynamic spirometry was done at all time points. Residual symptoms and health-related quality-of-life (HRQL) were evaluated using validated questionnaires.

**Results:**

At 10 months, most patients (81/118; 69%) were classified as high-ROG, of which 70% had severe ARDS during hospitalisation; 69% of those with mild-moderate ARDS also had high-ROG. Patients with high-ROG had longer ICU stay and lower PaO2/FiO2 during hospitalisation (*p* < 0.01). At 9 months follow-up, patients with high-ROG had smaller lung volumes as % of predicted values [mean (±CI): 80 (77–84) vs. 93 (88–98) (*p* < 0.001)], lower DLCO as % of predicted values [74 (70–78) vs. 87 (82–92) (*p* < 0.001)], lower oxygen saturation during 6MWT (*p* = 0.02), and a tendency to more severe dyspnoea (*p* = 0.07), but no difference was found in HRQL compared with no/minor ROG (*p* = 0.92). A higher opacity score was related to lower DLCO at follow-up (*r* = −0.48, *p* < 0.001, Spearman rank test). Severe ARDS patients had slightly more severe fatigue at 9 months compared to mild–moderate, but no differences in dyspnoea or lung function at follow-up. Fibrotic-like changes were found in 93% of patients examined with CT scans at 2 years (55/118; 47%). Severe ARDS could predict widespread opacities (ROG > 25%) in most patients at follow-up at 10 months (AUC 0.74).

**Conclusion:**

Residual radiological abnormalities in ICU-treated COVID-19 patients, evaluated for up to 2 years, relate to persisting symptoms and impaired lung function, demanding careful follow-up regardless of ARDS severity at hospitalisation.

## Highlights

- 70% of COVID-19 patients treated in the ICU had persisting widespread residual opacities at CT scans 10 months after hospital discharge.
- Residual opacities were associated with poorer spirometry results and lower oxygen saturation during 6MWT.
- A strong correlation was observed between lower lung diffusion capacity and residual opacities at a CT scan 10 months after hospital discharge.
- Patients with severe ARDS experienced greater fatigue at follow-up but showed no differences in lung function compared to mild–moderate ARDS.
- Fibrotic-like changes were found in 92% of patients examined (*n* = 55) on a 2 years follow-up.

## Introduction

During the first year of the COVID-19 outbreak, 7,058 people in Sweden were treated in intensive care (1). In previous coronavirus outbreaks with SARS and MERS, long-term radiological sequelae were reported (2), and a 15 years-long follow-up of severely ill SARS-infected health workers in Beijing, assessed with spirometry and computed tomography (CT) scans, showed persisting ground-glass opacities and lung function to be impaired in 38% of them (2).

There are discording reports on long-term follow-up after severe respiratory failure requiring intensive care unit (ICU) admission in COVID-19. While some studies have described the resolution of radiological sequelae within 1 year (3), other studies have reported persisting respiratory impairment 2 years after hospital discharge in patients treated with respiratory support and thus severely ill in the acute phase (4), including the presence of fibrotic-like changes at CT scan (5). Several smaller prospective studies that described radiology findings and lung function assessed from a few months up to 1 year after hospital discharge have been published, and these have a wide range of inclusion criteria and varying results (6–8). However, studies focused on both radiological and respiratory outcomes in survivors of severe, ICU-treated COVID-19 are relatively scarce (9–13), and more information is needed regarding the relevance of persistent radiological findings in this patient population.

Our primary aim was to investigate whether acute respiratory distress syndrome (ARDS) severity during the ICU stay predicted functional and respiratory outcomes assessed using spirometry, 6 min walk test, questionnaires, and CT scans performed during the first 2 years of follow-up. Our secondary aim was to investigate if more deranged clinical and laboratory data during hospitalisation and longer ICU stay were associated with worse results both in radiological outcomes in functional tests and in health-related self-evaluation at follow-up. Gender differences were also of interest. We hypothesised that ARDS severity would be associated with a higher degree of lung opacity and impaired physical performance at follow-up.

## Materials and methods

At the Karolinska Hospital in Stockholm, Sweden, a clinical multidisciplinary follow-up of critically ill COVID-19 patients has been ongoing since the spring of 2020, including functional testing and radiology at 6 months to 1 year intervals. Using follow-up data and data from hospital care records, we performed a single-centre prospective follow-up study of patients treated in the ICU from 19 March 2020 to 18 January 2021.

Vaccination was not available during this period, but all patients received standard low-molecular-weight heparin prophylaxis from the start of the study, with doses doubled from 14 April 2020. Patients admitted to the ICU during the study period were treated with the same strategy established during the pandemic by local guidelines.

**Figure 1:**
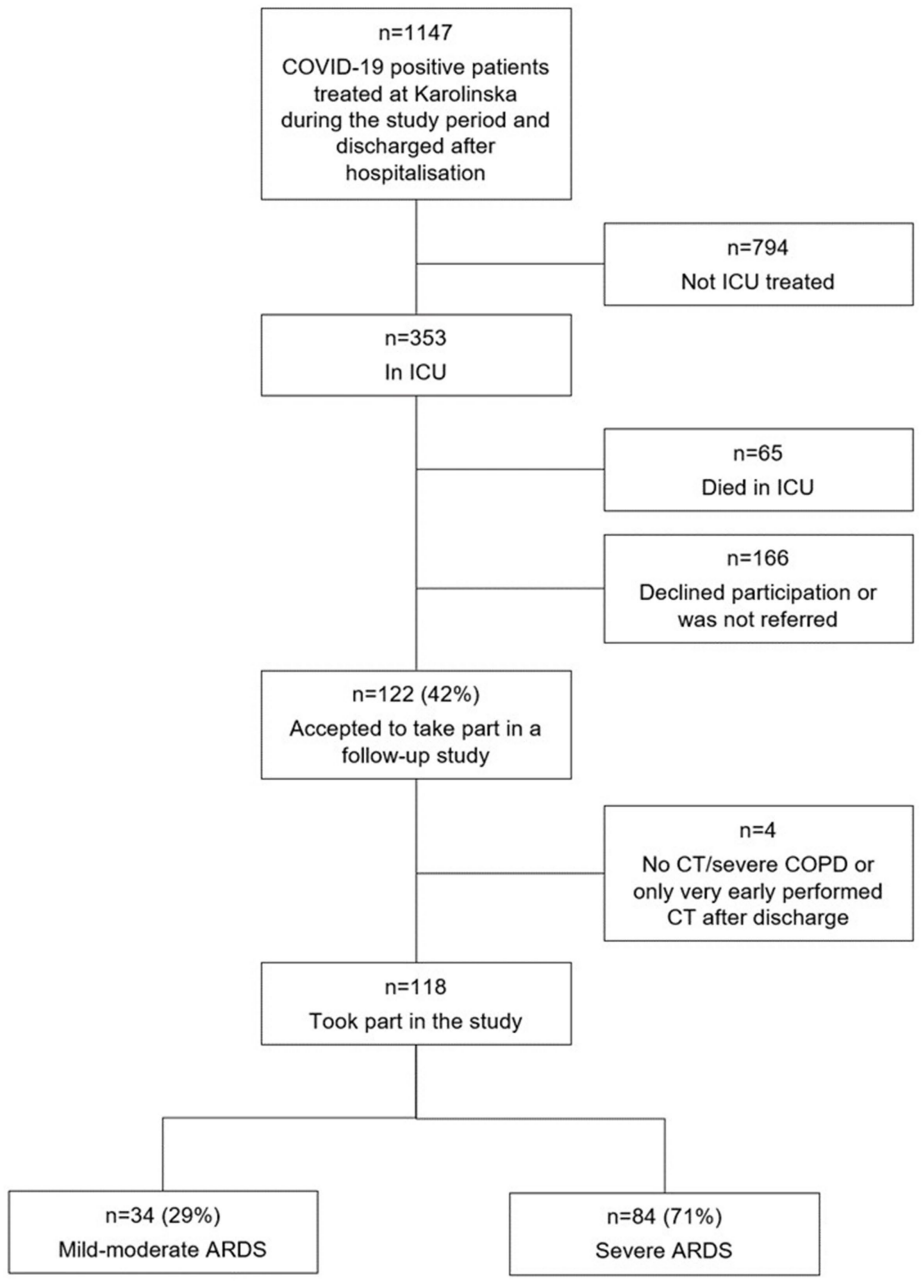
Flowchart of the patient selection.

During the study period, 353 COVID-19 patients were treated in the ICU. Of the surviving 288 patients, 122 patients agreed to participate in the present study, representing 42% participation (Figure 1). No patient in the study died during the first 15 months after discharge. A thoracic CT scan performed at least 5 months after hospitalisation was a prerequisite for participation, leading to the final inclusion of 118 patients in the analyses. One patient was excluded due to a preexisting lung condition, severe chronic obstructive pulmonary disease (COPD), and pulmonary fibrosis. Follow-up visits were planned at 3, 6, and 12 months but actually took place at 3, 9, and 15 months after discharge and were labelled accordingly.

Eleven patients (9%) received non-invasive ventilation (NIV) or high-flow nasal oxygen (HFNO) therapy only; the remainder were treated with invasive mechanical ventilation. Information on hospital and ICU lengths of stay, duration of mechanical ventilation, demographics, body mass index (BMI), and co-morbidities was collected from the electronic case records system (Tables 1, 2).

**Table 1:** Demographics and outcome data during ICU hospitalisation.

Demographics	Full cohort	Low-ROG group	High-ROG group	*p*-value	Mild-moderate ARDS	Severe ARDS	*p*-value
	(*n* = 118)	(*n* = 37)	(*n* = 81)		(*n* = 34)	(*n* = 84)	
Gender (male/female) (nr), *n* = 118	93/25	29/9	64/16	0.21	27/7	64/20	0.78
Age (years), *n* = 118	58 (51–64)	53 (44–60)	59 (52–66)	<0.001	58 (51–62)	58 (51–65)	0.48
Weight (kg)	87 (76–97)	90 (82–99)	87 (76–101)	0.57	90 (76–101)	89 (76–99)	0.71
Height (cm)	174 (166–180)	174 (169–180)	173 (166–180)	0.65	172 (169–182)	174 (166–189)	0.34
BMI (kg/m^2^)	30 (27–34)	30 (28–33)	30 (26–34)	0.45	30 (26–34)	30 (27–34)	0.88
Co-morbidities							
Hypertension *n* = 113, % yes (nr yes/no)	42 (43/70)	44 (16/20)	35 (50/27)	0.34†	38 (13/21)	38 (31/50)	1.0†
Cardiac disease *n* = 113	12 (14/99)	8 (3/33)	14 (66/11)	0.37†	9 (3/31)	14 (11/70)	0.48†
Diabetes, *n* = 68	29 (20/48)	48 (10/11)	21 (37/10)	0.028†	20 (4/16)	32 (16/34)	0.32†
COPD *n* = 113	3 (3/110)	3 (1/35)	3 (2/75)	0.96†	0 (0/34)	4 (3/78)	0.26†
Allergy *n* = 113	13 (15/98)	11 (4/32)	4 (3/78)	0.14†	9 (3/31)	6 (5/76)	0.61†
Asthma *n* = 112	12 (14/98)	22 (8/28)	8 (6/70)	0.032†	18 (6/28)	11 (9/71)	0.36†
Kidney disease *n* = 113	9 (10/103)	11 (4/32)	8 (6/71)	0.56†	15 (5/29)	6 (5/76)	0.14†
Thromboembolic disease *n* = 113	5 (6/107)	0 (0/36)	8 (6/71)	0.09†	3 (1/33)	7 (6/76)	0.36†
Cancer *n* = 113	7 (8/105)	6 (2/34)	8 (6/71)	0.67†	9 (2/31)	6 (5/75)	0.61†
Tobacco use *n* = 110, % yes (nr yes/no)	52 (57/53)	48 (16/17)	53 (41/36)	0.64†	58 (19/13)	49 (38/40)	0.26†

Demographics are expressed as median (interquartile range), co-morbidities are expressed as % affected, *n* = affected subjects in each group/not affected subjects. AKI acute kidney injury; ARDS, acute respiratory distress syndrome; BMI, body mass index; CI, confidence interval; COPD, chronic obstructive pulmonary disease. *p* < 0.01 was considered significant. Statistical analyses are based on analysis of variances; Student *t*-test and †Pearson chi-squared for categorical data.

**Table 2:** Laboratory data and outcome data during hospitalisation and radiology at 10 months.

Laboratory data	Full cohort	Low-ROG group	High-ROG group	*p*-value	Mild-moderate ARDS	Severe ARDS	*p*-value
CRP maximum during hospitalisation (mg/L)	325 (303–347)	284 (242–327)	344 (318–369)	0.014	319 (273–365)	328 (302–353)	0.73
D-dimer max during hospitalisation (mg/L)	7.2 (5.9–8.7)	4.4 (3.2–6.2)	9.0 (7.2–11.3)	<0.001	5.0 (3.3–7.5)	8.1 (6.6–10.1)	0.048
IL-6 max (pmol/L), *n* = 90	237 (102–520)	114 (74–176)	332 (244–453)	<0.001	144 (91–227)	269 (194–474)	0.05
PaO_2_/FiO_2_ (kPa)	11.0 (10.4–11.8)	12.6 (11.3–14.2)	10.6 (9.6–11.2)	0.003	16.6 (15.5–17.8)	9.4 (8.9–9.9)	<0.0001
Outcome data during ICU							
Time on mechanical ventilation	19 (16–21)	11 (8–14)	22 (19–26)	<0.001	12 (8–15)	21 (18–25)	<0.01
AKI, *n* = 118, % yes (and nr yes/no)	38 (45/73)	24 (9/28)	44 (36/45)	0.036†	21 (7/27)	45 (38/46)	0.79†
Renal replacement therapy, *n* = 118	22 (26/92)	14 (5/32)	25 (21/60)	0.13†	12 (4/30)	26 (22/62)	0.59†
Pulmonary embolism, *n* = 118	20 (24/94)	14 (5/32)	23 (19/62)	0.21†	12 (4/30)	24 (20/64)	0.15†
Pneumomediastinum/pneumothorax, % (nr yes, no)	8 (10/108)	0 (0/37)	12 (10/71)	0.025†	6 (2/32)	10 (8/76)	0.52
ECMO, %, (nr yes/no)	7 (8/108)	0 (0/37)	10 (8/73)	0.048†	0 (0/34)	10 (8/76)	0.08†
Hospital LOS (days)	39 (35–43)	26 (21–31)	44 (39–49)	<0.001	29 (23–35)	42 (37–47)	<0.01
ICU LOS (days)	21 (18–24)	13 (10–16)	25 (21–28)	<0.001	13 (10–17)	24 (12–34)	<0.001
Subjects with severe ARDS % yes (nr yes/no)	71 (84/34)	65 (24/13)	74 (60/21)	0.31			
Subjects classified as “High-ROG-group,” % yes (nr yes/no) at 10 months	69 (81/37)				62 (21/13)	71 (60/24)	0.31

Laboratory and outcome data during ICU as mean ± confidence intervals (CI). D-dimer, IL-6, and PaO_2_/FiO_2_ were not normally distributed and log-transformed and are expressed as geometric mean (±CI). Outcome data are expressed as % affected, *n* = affected subjects in each group/not affected subjects. *p* < 0.01 was considered significant. Statistical analyses are based on analysis of variances; Student *t*-test and †Pearson chi-squared for categorical data. CRP, C-reactive protein; CT, computed tomography; ECMO, extracorporeal membrane oxygenation; High-ROG, patients with more widespread opacity changes on 10 months CT; ICU, intensive care unit; IL-6, interleukin-6; LOS, length of hospital stay; Low-ROG, patients with no/minor opacities in 10 months CT; PaO_2_/FiO_2_, quotient between arterial partial oxygen pressure and fraction of inspired oxygen.

### Ethical considerations

The study was approved by the Swedish Ethical Review Board (Application number 2020-02394, 2020-02149, and 2021-04735), and written informed consent was obtained.

### Measurements

Highest values for C-reactive protein (CRP; reference <5.0 mg/L), D-dimer (reference <0.5 mg/L for <50 years and <0.7 mg/L for >70 years), and interleukin (IL)-6 (reference value <17.4 pg./mL) during hospital care were retrieved.

To indicate the acute phase disease severity, the PaO_2_/FiO_2_ ratio was recorded at its lowest value during the first 24 h after intubation in the ICU. Severe ARDS syndrome was defined by a PaO_2_/FiO_2_ ratio of ≤13.3 kPa and mild-moderate ARDS by PaO2/FiO2 < 40 kPa. The PaO_2_ was obtained from blood gas analysis (ABL800 FLEX, Triolab AB, Mölndal, Sweden).

### Radiology after discharge

#### CT investigation

Radiological information was retrieved from databases. Patients were examined at the 3- and 6 months follow-up visits, respectively, at 3 (*n* = 21), 4–7 months (*n* = 75), and 8–12 months (*n* = 96). A late investigation at 20–25 months was performed at clinician discretion in 55 patients. Below, the timing of CT follow-up has been simplified and reported as conducted at approximately 4, 10, or 22 months after discharge. Of the patients who underwent the 10 months control, 22 were also examined at 5–7 months. Results from 4 months are presented from the subjects with a CT scan at 22 months.

#### CT protocol

All CT scans were analysed similarly using a 256-slice multi-detector CT (Siemens Healthineers). The scans were analysed using 0.63 mm slice reformats in orthogonal planes.

#### Evaluation of images

Two independent radiologists analysed the scans at 10 months and were blinded to hospitalisation details and follow-up results.

The severity of pulmonary parenchymal involvement was classified and graded regarding ground-glass opacities, consolidations, reticular and crazy-paving patterns, and the presence of airway and pleural changes, nodules, and some other less common findings (9). Pulmonary findings were semi-quantified according to the Radiological Opacity Score (ROG). The ROG represents an estimated mean opacity value of parenchymal abnormalities, which was calculated for each patient by summing the values for all lobes and dividing them by five (the total number of lobes). The middle lobe values were given half the weight of the other four lobes (14). The patients were grouped by opacity extension at 10 months in either the low-ROG group with no or only minor opacities on CT (up to 5%, *n* = 37) or in the high-ROG group with more widespread opacities (>5%, *n* = 81) and moderate-to-severe opacities.

In 11 cases (9%), the two radiologists had diverging opinions regarding the extent of opacifications. In these cases, a third radiologist, who was also blinded, re-examined the scans and reached a consensus.

Pulmonary findings in each lobe were further classified by one radiologist (5, 15) rounded up into percentage coverage, as follows: 0, 5, 10, 20, 35, and 60%.

From these findings, the cohort was further grouped into four categories according to the 10 months ROG: normal/minor, with no or minor changes on thoracic CT (<5%, *n* = 37); moderate changes (5–15%, *n* = 22), severe changes (15–25%, *n* = 40), and widespread severe residual changes (>25%, *n* = 19). These categories were used to track associations of opacity severity with functional outcome tests.

At 22 months, CT scans were also examined to determine the prevalence of fibrosis-like abnormalities.

The main pulmonary artery diameter was also measured proximally to the pulmonary artery bifurcation on an axial slice, vertical to its long axis, using 2 mm slice formats.

### Follow-up after discharge

Dynamic spirometry was performed at the 3 months visit and spirometry, including body plethysmography and diffusion capacity for carbon monoxide (DLCO), at 9- and 15 months visits. Spirometry results were expressed as a percentage of predicted values, using reference values for the Swedish population (16, 17). Diffusion capacity was measured using standardised procedures (18).

A 6 min walk test (6MWT) was performed at the 3- and 9 months visits (19). Pulse oximetry was recorded before and during the test. The BORG CR-10 scale and RPE6-20 scale were used to evaluate persisting symptoms at follow-up (20). The COPD assessment test (CAT) (21) and EQ-5D protocol (22) were used to validate residual symptoms and current health at the 3- and 9 months visits.

### Statistical analysis

The anthropometric data and radiological parameters are presented as medians with interquartile ranges (IQRs). Laboratory parameters, as well as spirometry and 6MWT results, are presented as means and confidence interval ranges (±CI). Normal distribution was achieved by log-transformation of D-dimer, IL-6 data, and PaO_2_/FiO_2_, presented as geometric means (±CI). Student’s *t*-test and ANOVA were used to compare means when the distribution was normal; otherwise, the Mann–Whitney U-test, Kruskal–Wallis ANOVA, Wilcoxon, and Spearman rank test were used. Pearson’s *x*^2^ test was used to compare categorical variables.

Comparisons were performed between low- and high-ROG groups and the mild–moderate versus severe ARDS patients. The cohort was also stratified into four categories according to the 10 months opacity score.

**Figure 2:**
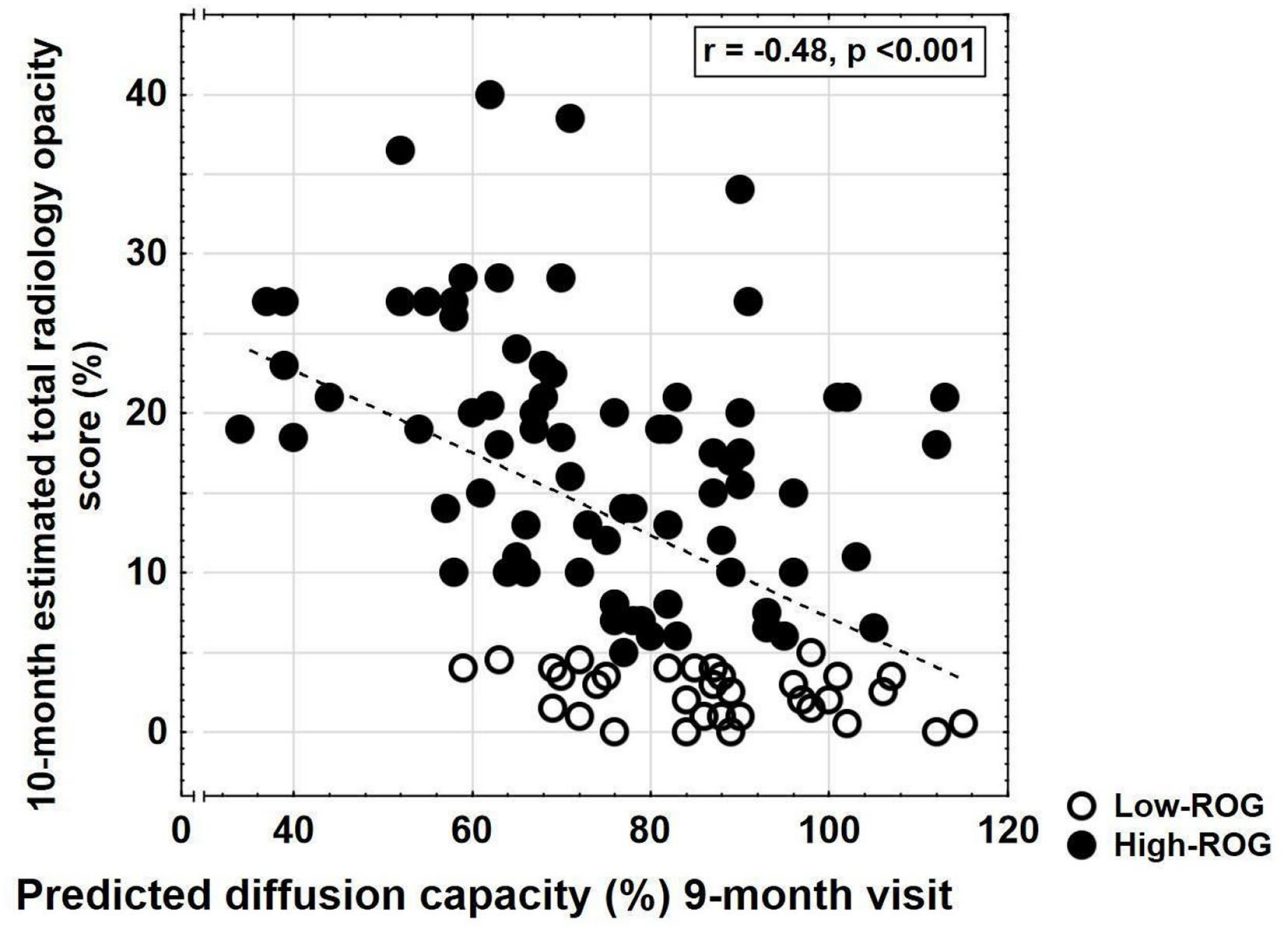
Relationship between radiology opacity score at the 10 months follow-up and predicted diffusion capacity at spirometry at the 9 months visit. Subjects with low-ROG (unfilled black dotted) with no/minor residual changes and high-ROG (unfilled dotted) with more widespread changes are shown. A significant relationship was found (*r* = −0.48, *p* < 0.001, non-parametric Spearman rank test).

The predictive efficacy of ARDS severity for DLCO ≤60% and ROG (higher than 5% and higher than 25%, respectively) was evaluated by receiver operating characteristic (ROC) curves (Figure 2). Linear regression analysis was performed to compare PaO_2_/FiO_2_ values to spirometry outcomes, i.e., DLCO at 9 months. The non-parametric Spearman’s rank correlation assessed the relationship between DLCO at the 9 months CT scan and estimated global radiology opacity percentage at the 10 months CT scan. To investigate the impact of different outcomes, odds ratio analysis was performed with the “moderately reduced 9 months DLCO” (DLCO <60%) (23) as the dependent variable. Upper or lower limits of IQR values for the respective variables were used in the analysis, except for total hospital stay and ICU stay, for which median values were used.

A *p*-value of <0.01 was considered significant; 0.01 ≥ *p* < 0.05 was considered a tendency. Statistical analyses were performed using Statistica (version 14; StatSoft, Tulsa, OK, United States).

## Results

### Demographic and clinical findings during hospitalisation

Table 1 presents demographic data and co-morbidities, and Table 2 presents the laboratory and outcome data for the cohort during the ICU stay, along with the results from the comparisons between low- versus high-ROG groups and the mild-moderate versus severe ARDS groups.

Most patients were men (79%), with a median age of 58 years (Table 1). Hypertension and diabetes were common co-morbidities. Fifty-two percent of the patients were active/previous smokers. The mean time on mechanical ventilation was 19 days, and the mean hospitalisation time was 39 days. Twenty-two percent needed renal replacement therapy and 7% ECMO. Pulmonary embolism (PE) was found in 20%.

The mean PaO_2_/FiO_2_ was 11 kPa, indicating that most patients had severe ARDS. One subject was classified as mild ARDS (PaO_2_/FiO_2_ > 26 kPa). The mean PaO_2_/FiO_2_ in ECMO patients (*n* = 8) was 6.4 kPa; in those who received NIV (*n* = 11), it was 13.7 kPa (Table 2).

### Relationship between pulmonary radiology at follow-up and clinical findings during hospitalisation

The median age in the low-ROG group was significantly lower than in the high-ROG group (Table 1).

**Figure 3:**
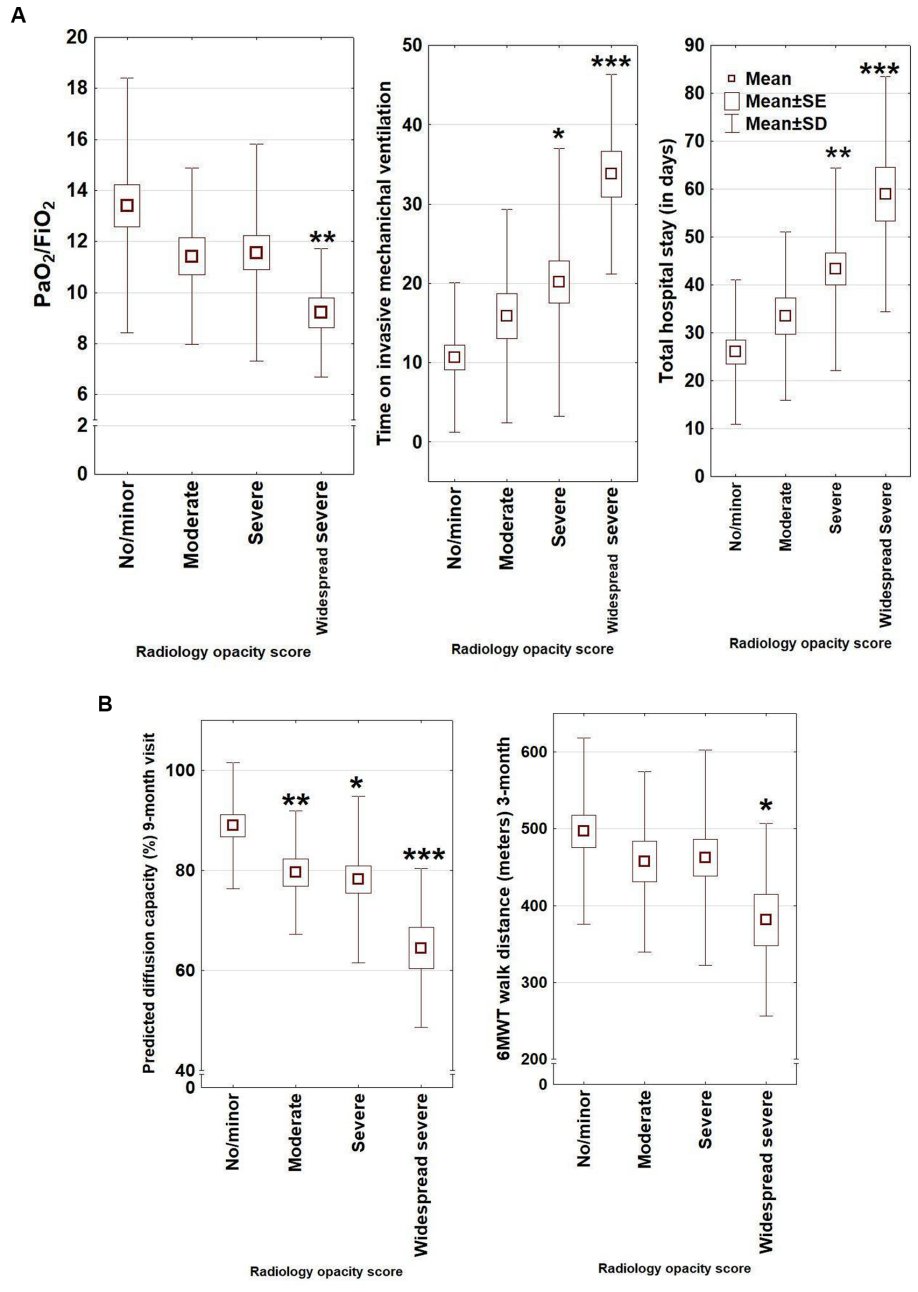
**(A)** A significant association with longer time on mechanical ventilation, longer hospital stay, and lower PaO2/FiO2 during hospitalisation was present (Kruskal–Wallis test) in patients with widespread severe remaining opacities compared with no/minor residual opacities at the 10 months CT scan (Kruskal–Wallis test and according to multiple comparisons of mean ranks for all groups). Dots present mean, box mean +/− SE, and bars mean +/− SD for different variables (*p < 0.05, **p < 0.01, and ***p < 0.001). **(B)** Radiological opacity stratified as no/minor, moderate, severe, and widespread/severe at 10 months. In the widespread/severe opacity group, a lower predicted diffusion capacity at 9 months and shorter 6MWT distance were present at follow-up, compared with the no/minor opacity group (Kruskal–Wallis test and multiple comparisons of the mean rank test, *p < 0.05, **p < 0.01, and ***p < 0.001). For DLCO, the widespread/severe group differed significantly from all other respective groups (*p* less than or equal to 0.01). Dots present mean, box mean +/− SE, and bars mean +/− SD for different variables.

During hospitalisation, inflammatory markers, as well as the D-dimer, were significantly more elevated in the high-ROG group, while the oxygenation (PaO_2_/FiO_2_) was significantly lower in the same group. All barotrauma cases (pneumothorax/pneumomediastinum) were also in this group. There was a clear association between more severe radiology opacities and lower oxygenation, longer mechanical ventilation, and prolonged hospitalisation (Table 2 and Figure 3A).

### ARDS severity in ICU

D-dimer and IL-6 trended to be higher, and time spent on mechanical ventilation and length of ICU and hospital stay were longer in the severe ARDS group (Table 2).

### Radiology at follow-up

At 10 months after discharge, most patients (69%) were classified as high-ROG (>5% opacity), indicating more widespread and persistent lung disease, independently of the ARDS severity. Subjects with normal CT at 10 months (6%) were slightly younger compared with the rest (*p* = 0.01, all <60 years) (Table 2).

**Figure 4:**
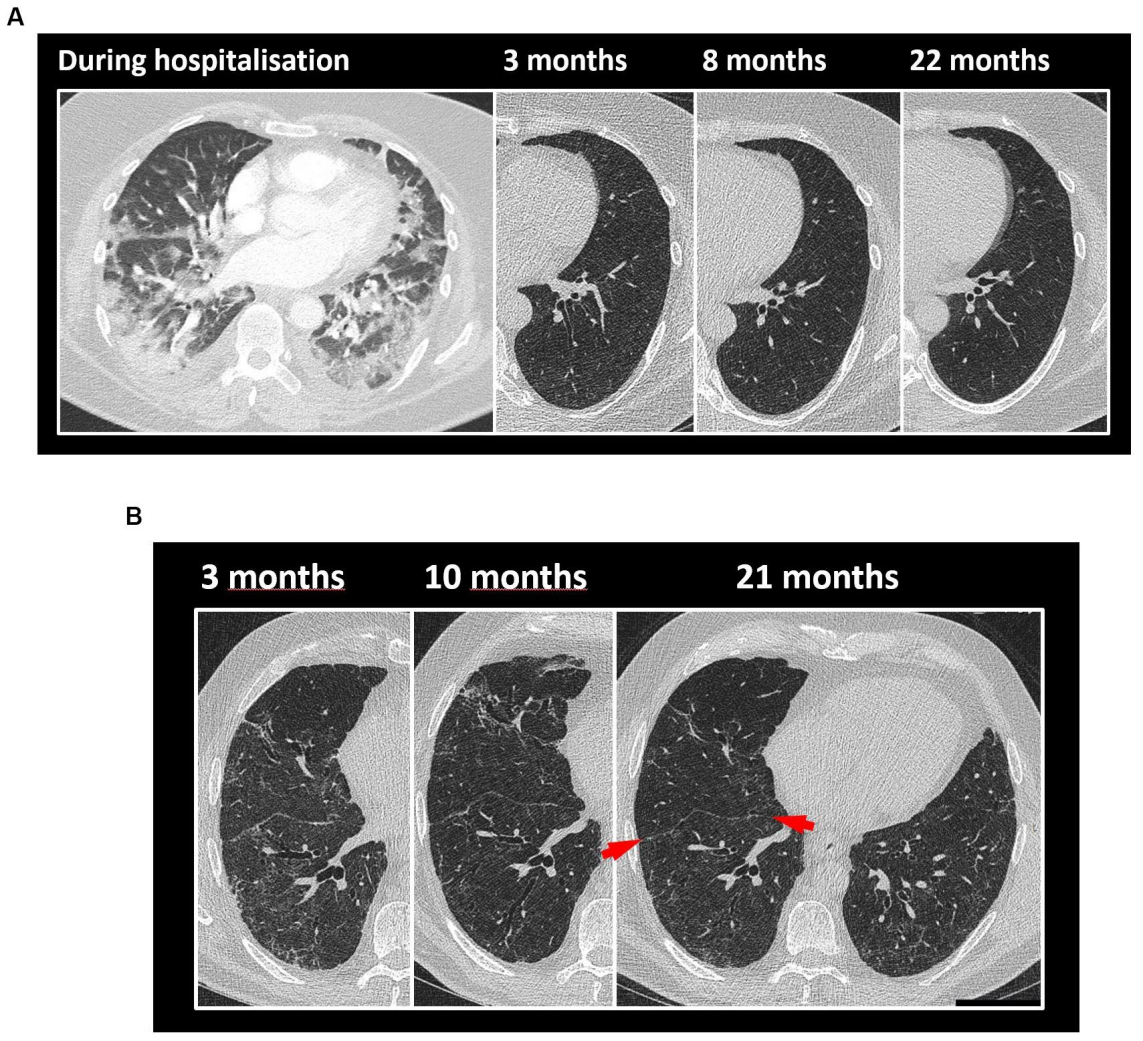
The temporal evolution of CT changes in two cases is illustrated, classified as low- and high-ROG, respectively. **(A)** Low-ROG. A woman in the mid-fifties was classified as low-ROG with no or minor residual opacities at the 10 months CT follow-up. The CT images, from left to right, depict her status during hospitalisation at 3 months, 8 months, and 22 months follow-ups. In the total cohort, 31% were classified as having no to minor opacities approximately 10 months after discharge. Most patients, both in the low- and high-ROG groups, exhibited air-trapping at the 10 months follow-up. **(B)** High-ROG. A man in the mid-sixties was classified as high-ROG with more widespread residual opacities on thoracic CT at the 10 months follow-up. CT images from left to right at 3 months, 10 months, and 21 months follow-ups. As an indication of an underlying fibrotic process, the interlobular fissure appears distorted (red arrow). At the 2 years follow-up, the majority displayed fibrotic-like changes in the parenchyma, including reticulation, bronchial dilatation, and distortion. Subpleural bands were also relatively common features at the 2 years follow-up.

In Table 3, the radiologic opacity scores from subjects studied up to 22 months (*n* = 55) are shown. A significant improvement was present between the 4- and 10 months follow-up (*p* < 0.01, Wilcoxon matched paired test), which was not observed between 10- and 22 months follow-up (*p* = 0.26). At 22 months, most patients (93%) had persistent fibrotic-like changes. Figure 4 illustrates the temporal evolution of CT changes in two cases, classified as low- and high-ROG, respectively.

**Table 3:** Data from follow-up, radiology.

Follow-up	Subjects studied at 22 months follow-up *n* = 55
Radiologic morphology at CT	
1. At 4 months	
Months after hospital admission	4 (4–5)
Global radiology opacity percentage *(%)	20 (10–26)
Main pulmonary artery diameter (mm)	29 (27–30)
2. At 10 months	
Months after hospital admission	10 (8–11)
Global radiology opacity percentage (%)	15 (6–21)
Right upper lobe (Radiology opacity percentage)	20 (10–35)
Middle lobe	20 (10–20)
Right lower lobe	10 (5–35)
Left upper lobe	10 (5–20)
Left lower lobe	10 (5–20)
Main pulmonary artery diameter (mm)	29 (28–30)
2. At 22 months	
Months after hospital admission	22 (18–25)
Global radiology opacity percentage (%)	15 (5–20)
Main pulmonary artery diameter (mm)	29 (28–30)
Normal CT scans, % yes (nr yes/no)	7 (4/55)

Values are expressed: demographics and radiology outcome median (interquartile range), main pulmonary diameter as mean (±CI). See material and methods for estimation.

### Clinical follow-up

Table 4; Supplementary Tables S1, S2 show spirometry outcome, demographic and clinical data at follow-up for the full cohort and groups based on the ROG score, and ARDS severity.

**Table 4:** Data from spirometry outcome.

Follow-up	Full cohort	Low-ROG group	High-ROG group	*p*-value	Mild-moderate ARDS	Severe ARDS	*p*-value
SPIROMETRY 3 months visit, *n* = 109‡							
FVC (L) % of predicted values	69 (66–73)	76 (71–81)	66 (62–70)	0.005	73 (56–77)	68 (63–72)	0.14
FEV_1_ (L) % of predicted values	71 (68–87)	76 (71–81)	69 (64–73)	0.065	74 (70–78)	69 (65–74)	0.18
FEV_1_/FVC ratio expected values	103 (100–106)	98 (91–105)	106 (103–109)	0.012	103 (98–111)	103 (100–107)	0.87
Max inspiratory pressure (cmH_2_O)	90 (84–96)	103 (92–114)	84 (77–91)	0.003	90 (78–102)	90 (83–98)	0.96
Max expiratory pressure (cmH_2_O)	96 (86–106)	104 (84–125)	92 (81–103)	0.24	102 (83–120)	93 (81–105)	0.40
Dynamic, 9 months visit *n* = 110							
Time after discharge (months)	8.3 (6.7–9.2)	8.5 (6.7–10)	8.2 (6.7–9.1)	0.73	8.2 (6.8–9.6)	8.4 (6.6–9.2)	0.28
FVC (L) % of predicted values	84 (81–87)	89 (83–94)	82 (78–86)	0.044	84 (79–89)	84 (80–88)	0.98
FEV_1_% of predicted values	86 (83–89)	86 (80–91)	86 (82–89)	0.95	85 (79–89)	86 (82–89)	0.88
FEV_1_/FVC ratio expected values	104 (91–117)	91 (87–95)	110 (91–130)	0.18	104 (96–111)	97 (94–101)	0.11
Total lung capacity (L) % of predicted values	84 (81–88)	93 (88–98)	80 (77–84)	<0.001	85 (80–90)	84 (80–88)	0.69
Diffusion capacity % of predicted values, *n* = 108	78 (74–81)	87 (82–92)	74 (70–78)	<0.001	82 (78–87)	76 (72–80)	0.09
Dynamic, 15 months visit *n* = 51, Time after discharge (months)	15 (13–18)	18 (13–26)	14 (13–16)	0.10	15 (13–18) (*n* = 11)	15 (13–19) *n* = 40	0.81
FVC (L) % of predicted values	83 (79–88)	88 (78–98)	81 (76–86)	0.19	84 (76–91)	82 (76–88)	0.79
FEV_1_% of predicted values	84 (72–94)	86 (73–99)	83 (78–88)	0.61	85 (74–95)	83 (78–89)	0.76
FEV_1_/FVC ratio expected values	104 (101–107)	100 (91–109)	104 (101–108)	0.27	103 (95–111)	104 (100–107)	0.96
Total lung capacity (L) % of predicted values	85 (81–90)	96 (88–105)	83 (78–87)	<0.01	87 (78–95)	85 (80–90)	0.79
Diffusion capacity (DLCO)% of predicted values	75 (69–81)	85 (73–97)	76 (71–81)	0.43	80 (72–88)	74 (66–81)	0.39

Data are expressed as mean (±CI). *p* < 0.01 was considered significant. Statistical analyses are based on analysis of variances and student *t*-test. ARDS, acute respiratory distress syndrome; FEV_1_, forced expiratory volume in 1 s; FVC, forced vital capacity; High-ROG, patients with more widespread opacity changes on the 10 months CT scan; Low-ROG, patients with no/minor opacities in the 10 months CT scan.

Spirometry showed a mild reduction in forced vital capacity (FVC) (69% of normal) but no signs of airway obstruction. DLCO at the 9- and 15 months follow-up was 78 and 75% of predicted values (Table 4); 21% of the subjects showed normal total lung capacity and 51% had ordinary (>75%) DLCO at 9 months. Patients with PE during hospitalisation had lower DLCO at 9 months, with a mean predicted value of 67% versus 80% (*p* < 0.01) in patients without PE.

**Figure 5:**
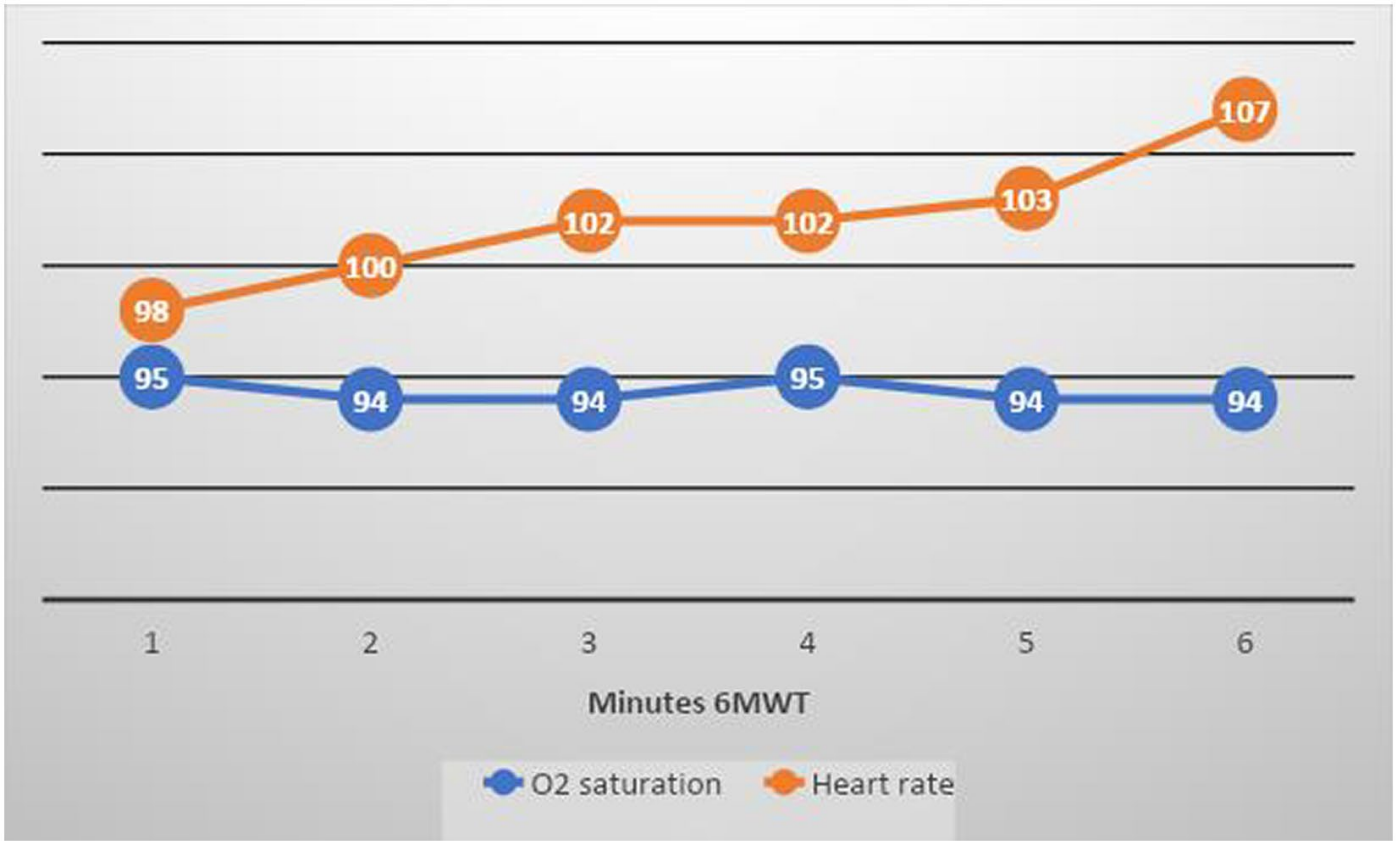
Mean O 2 saturation and heart rate at 3 months follow-up 6MWT in the full cohort (blue line shows O 2 saturation and orange line shows heart rate). The test was submaximal, with only a small increase in mean pulse rate (around 10 beats/min).

Table 5 shows clinical data from the 6MWT test. The 6MWT distance increased between 3- and 9 months follow-up from 85 to 97% of reference values. The test was submaximal, with only a small increase in median pulse rate (around 10 beats/min) and a self-estimated breathlessness and fatigue of 3 on the BORG 1–10 scale (Table 5; Supplementary Table S2 and Figure 5).

**Table 5:** Data from follow-up and 6MWT outcome.

Follow-up	Full cohort	Low-ROG group	High-ROG group	*p*-value	Mild-moderate ARDS	Severe ARDS	*p*-value
6 min WALK TEST, *n* = 112, 3 month visit							
Time after discharge (days)	2.9 (2.4–3.7)	3.0 (2.4–4.1)	2.9 (2.3–3.6)	0.12	2.6 (2.1–3.5)	2.9 (2.4–3.7)	0.37
Distance (meters)	462 (437–487)	497 (450–564)	445 (413–477)	0.027	477 (433–521)	455 (424–487)	0.33
Distance of ref. (%)#	85 (79–91)	89 (79–99)	83 (66–96)	0.46	81 (74–88)	87 (79–87)	0.39
SpO_2_ at 1 min (%)	95 (95–96)	96 (96–97)	95 (98–100)	0.01	96 (95–97)	96 (95–96)	0.99
Heart rate at 1 min (beats/min)	98 (94–101)	101 (94–107)	97 (92–102)	0.35	95 (87–104)	99 (86–113)	0.27
SpO_2_ at 6 min (%)	94 (94–95)	96 (95–97)	95 (95–96)	<0.001	95 (94–96)	94 (93–95)	0.13
Heart rate at 6 min (beats/min)	107 (102–111)	110 (102–118)	105 (100–111)	0.30	102 (93–112)	111 (97–123)	0.21
9 months visit 6 MWT, *n* = 46							
Time after discharge (days)	10 (9–12)	11 (10–12)	10 (9–12)	0.63	10.5 (9–12)	10 (9–12)	0.38
Distance (meters)	505 (468–540)	520 (433–570)	488 (439–538)	0.23	481 (406–556)	518 (476–559)	0.36
Distance of ref. (%)#	97 (85–109)	95 (76–115)	98 (97–99)	0.84	80 (69–90)	106 (89–122)	<0.05
SpO_2_ at 1 min (%)	98 (98–99)	99 (98–100)	93 (87–100)	0.32	99 (97–100)	98 (97–99)	0.40
SpO_2_ at 6 min (%)	97 (96–98)	99 (98–100)	96 (95–98)	0.02	99 (98–100)	96 (95–198)	0.012

#According to the formula, see the material and methods section. Time after discharge is expressed as median (interquartile range). 6MWT outcome data are expressed as mean (±CI). 6MWT, 6-min walk test; High-ROG, patients with more widespread opacity changes on the 10 months CT scan; Low-ROG, patients with no/minor opacities in the 10 months CT; Min, minutes; Ref, reference; SpO2, oxygen saturation. *p* < 0.01 was considered significant. Statistical analyses are based on analysis of variances; Student *t*-test and ††Mann–Whitley U-test for non-parametric values.

Oxygen saturation during the 6MWT at 3 months was slightly reduced but normalised at 9 months.

Table 6; Supplementary Table S2, with data from CAT and EQ 5, show that breathlessness when walking on stairs (grades 4–5) was initially reported by almost half the total cohort (45%) but only by 26% at 9 months.

**Table 6:** Data from follow-up, questionnaires (CAT and EQ 5).

Perceived symptoms at 3 months visit	Full cohort	Low-ROG group	High-ROG group	*p*-value	Mild-moderate ARDS	Severe ARDS	*p*-value
CAT total score	14 (922)	13 (6–20)	15 (10–23)	0.13††	15 (11–21)	14 (8–22)	0.76††
EQ-5D total score	60 (50–75)	65 (52–80)	60 (50–65)	0.24††	60 (50–75)	65 (50–75)	0.96††
Very severe breathlessness (grade 5), % yes (nr yes/no)	21 (22/84)	10 (3/28)	25 (19/56)	0.07†	28 (8/21)	18 (14/63)	0.23†
Severe fatigue (grades 4–5), % yes (nr yes/no)	26 (27/78)	23 (7/24)	27 (20/54)	0.63†	21 (6/23)	28 (21/55)	0.47†
Perceived symptoms visit at 9 months visit							
CAT total score	10 (6–17)	9 (4–13)	10 (7–17)	0.30††	11 (7–13)	10 (6–20)	0.66††
EQ-5D total score	72 (50–82)	75 (50–85)	72 (55–85)	0.92††	70 (55–80)	74 (50–85)	0.71††
Severe breathlessness (grades 4–5), % yes (nr yes/no)	26 (23/67)	21 (6/23)	28 (17/44)	0.47†	28 (8/21)	25 (15/46)	0.76†
Severe fatigue (grades 4–5), % yes (nr yes/no)	16 (14/75)	14 (4/25)	17 (10/50)	0.73†	4 (1/27)	21 (13/48)	0.032†

The figures represent % (nr) of patients stating the symptoms of breathlessness and fatigue in grades ranging from 0 (none) to 5 (severe). For severe breathlessness and severe fatigue expressed as % affected, *n* = affected subjects in each group/not affected subjects. Breathlessness grading according to grade: 0 = “I have no breathlessness when walking in stairs or up a hill,” grade 5 = “When walking in stairs or up a hill I get severe breathlessness.” Fatigue grading according to grading, grade: 0 = “I have lots of energy,” grade 5 = “I do not have any energy.” ARDS, acute respiratory distress syndrome; CAT, COPD assessment test; EQ-5, standardised health quality of life questionnaire; High-ROG, patients with more widespread opacity changes on the 10 months CT scan; IQR, interquartile range; Low-ROG, patients with no/minor opacities in the 10 months CT scan. Scores presented as median (IQR). *p* < 0.01 was considered significant. Statistical analyses are based on †Pearson chi-squared for categorical data. ††Mann–Whitley U-test for non-parametric values.

### Relationship between radiology and functional tests at follow-up

**Figure 6:**
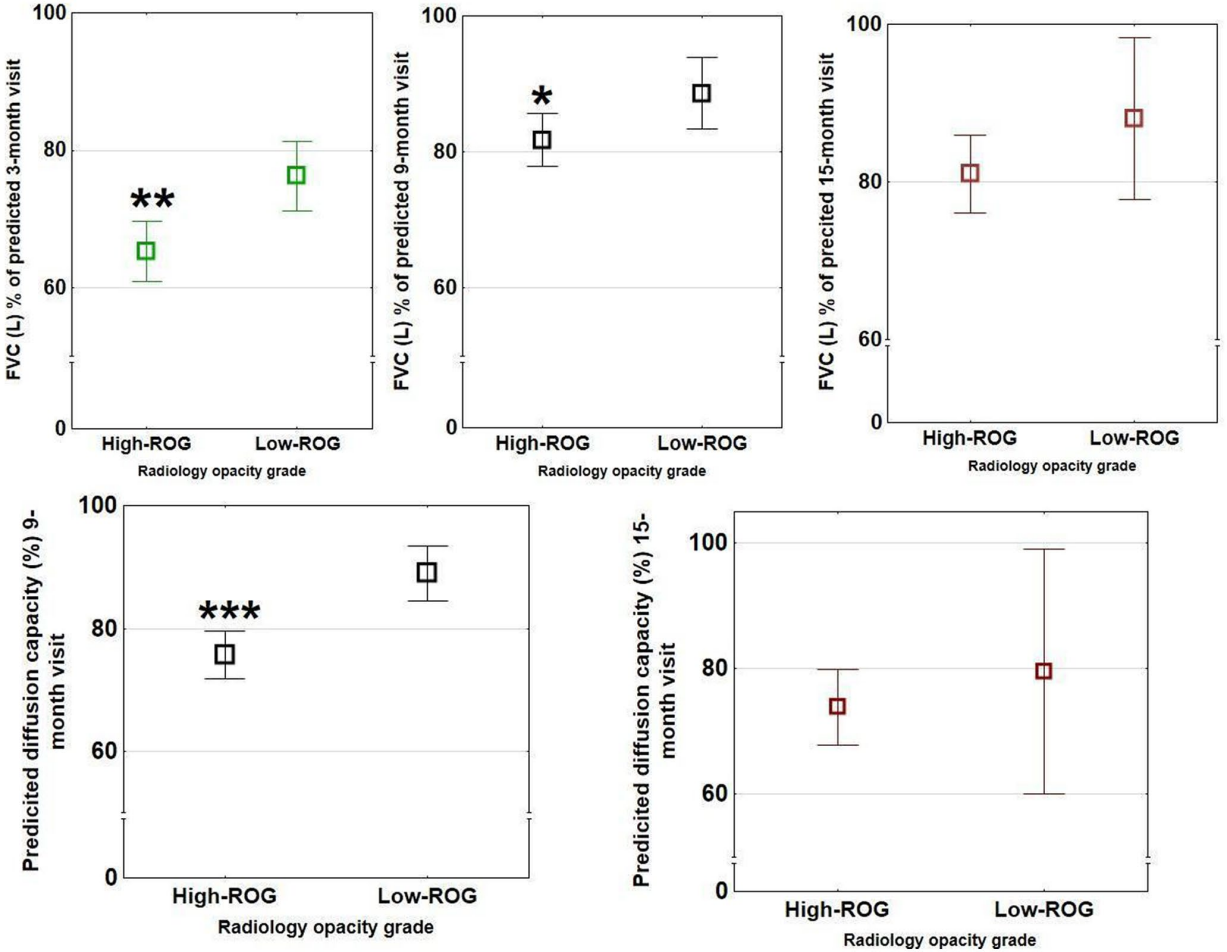
Spirometry outcomes in the two opacity groups at 3- (green bars), 9- (black bars), and 15- (red bars) month follow-up. Significant differences between ROG groups (low vs. high) were present for FVC at 3- and 9 months visits but not 15 months and for predicted diffusion capacity at 9 months (*n* = 111) but not 15 months (*n* = 50). These differences were not seen in the different ARDS groups. The rectangular boxes show means with bars showing confidence interval ranges (*p < 0.05, **p < 0.01, and ***p < 0.001).

The high-ROG group had lower maximal inspiratory pressure (MIP) at the 3 months follow-up, lower FVC at the 3- and 9 months follow-up, lower residual volume and total lung capacity at 9- and 15 months follow-up, and lower DLCO at the 9 months follow-up, compared with the low-ROG group (Table 4; Supplementary Table S1 and Figure 6). FEV1/FVC ratio was normal in both ROG groups.

The mean 6MWT distance trended to differ between ROG groups at 3 months follow-up. Oxygen saturation during the test showed a significant difference between groups and related to lung parenchymal residual pathology.

Patients with severe radiological opacities (when stratified into the four opacity categories) had lower DLCO and 6MWT (Figure 3B) but did not differ in CAT or total EQ-5 score. A significant relationship was found between higher radiological opacity at 10 months and lower DLCO at 9 months follow-up (Spearman rank test *r* = −0.48, *p* < 0.001, Figure 2).

### Relationship between ARDS severity and functional tests at follow-up

**Figure 7:**
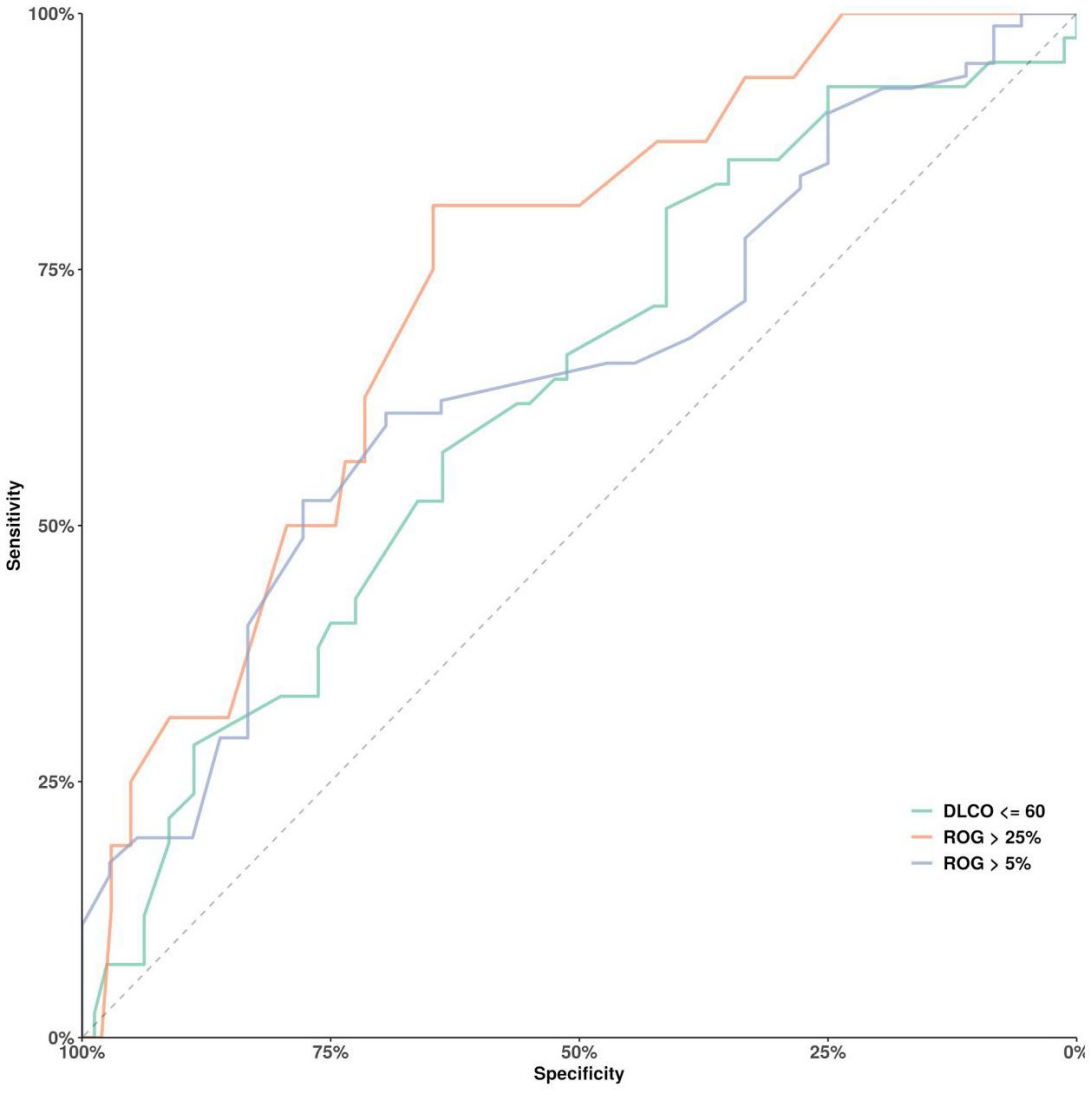
ROC curves for the predictive efficacy of the severe ARDS criteria (PaO2/FiO2 ≤ 13.3 KPa). The severe ARDS curves on a DLCO ≤60% at follow-up (AUC 0.64) (green line), on a high ROG (ROG >5%; AUC 0.65) (blue line), and on very high ROG (ROG >25%; AUC 0.74) (red line) at follow-up.

The two ARDS severity groups did not differ in spirometry, 6MWT outcomes, or EQ-5 scores (Tables 4–6; Supplementary Tables S1, S2). However, severe ARDS was associated with severe fatigue at follow-up. A positive correlation was observed between logarithmic PaO_2_/FiO_2_ at hospitalisation and DLCO at 9 months follow-up (*r* = 0.29, *r*^2^ = 0.09, *p* < 0.01) and was lower in women (Figure 1B). Severe ARDS showed an association that could better predict widespread severe radiology abnormalities (ROG >25%) at follow-up (AUC 0.74), not seen compared with ROG >5% (AUC 0.65) and/or DLCO ≤60% (AUC 0.64) (Figure 7).

### COVID-19 patients treated in ECMO

Patients treated with ECMO were younger (*p* < 0.01) (Supplementary Table S3). At follow-up, all ECMO patients were classified as high-ROG. Compared with the rest of the cohort, they had a lower DCLO (Figure 1A) and trended to a shorter 6MWT distance (*p* = 0.013) (Supplementary Table S3).

### Odds ratio analyses

Univariate OR analysis for certain variables for reduced DLCO (below <60%) at 9 months is presented in Supplementary Table S4. In multiple OR testing (with variable hospital stay and gender), a hospital stay longer than 19 days [OR = 33.13 (CI 3.41–321.9)] and female gender [OR = 8.30 (CI 1.75–39.27)] were both associated with DLCO below 60% (*p* < 0.001, *n* = 107).

## Discussion

The present study describes the radiological and functional follow-up of a cohort of COVID-19 patients hospitalised and admitted to the ICU during the first year of the pandemic when vaccines and antivirals were not available. Patients treated during the first year are of interest because their disease progression illustrates the natural course of the disease uninfluenced by vaccination status or antiviral treatments.

All patients had severely compromised lung function in the acute phase, with 71% having severe ARDS; 91% were treated with invasive ventilation and 7% even received ECMO treatment. All of them were discharged alive.

The primary aim of our study was to investigate if ARDS was associated with worse functional outcomes. In our population, severe ARDS was associated only with more fatigue at follow-up.

The secondary aim was to study if radiologic findings and functional outcomes were correlated. Our findings show that residual pulmonary opacities are associated with reduced lung volumes, shorter 6MWT distance, lower lung diffusion capacity, and dyspnoea at follow-up.

Widespread radiological opacities persisted in 70% of patients 10 months after hospital discharge. The radiological abnormalities did, however, decrease over time, and progression was only found in a minority of patients after 1 year. Patients with residual widespread radiological pulmonary opacities also had higher inflammatory parameters, prolonged mechanical ventilation, and hospital stay during the acute phase, all reflecting more severe acute disease.

Although radiological sequelae in COVID-19 are largely described as resolving within 1 year in patients without ARDS (3), a previous study including patients with ARDS to only a minor degree showed that 82% had remaining pathology at 2 years follow-up (4), a finding that is relatively consistent with our results. Those in our cohort who performed a 22 months follow-up improved radiologically at 10 months but were relatively unchanged or worsened at 2 years, showing in many cases “fibrotic-like” changes but no honeycombing. Previous reports describe at the 2 years follow-up the presence of fibrotic-like changes, such as reticulation, bronchial dilatation, and distortion (24, 25). Honeycombing is often considered specific to pulmonary fibrosis and represents damaged lung tissue containing cystic air spaces with thick fibrotic walls, usually localised in the peripheral parts of the lungs (26). Our radiological findings imply that major improvements are most likely to occur within the first year of hospital discharge. COPD or asthma were not associated with more severe pulmonary opacities in our study. Long-term studies are needed to investigate the risk of developing aggravated pulmonary fibrosis in this patient group and identify patients who may benefit from specific anti-fibrotic treatment (27, 28).

Lung volumes and ventilatory performance (reduced inspiratory muscle strength, measured as lower MIP) (29) were decreased in our entire cohort, and this reduction was more pronounced in the patient group with widespread opacities, indicating a persistent post-COVID-19 effect at 15 months. Previous follow-up studies on COVID-19 have shown differences in lung volumes in ICU patients compared with non-ICU inpatients (30), suggesting that such differences could be related to disease severity. Of our entire ICU cohort, 80% had reduced lung volumes at 9 months follow-up, in contrast to Orzes et al., who observed that approximately one-third of their patients had abnormal findings at 6 months (31). However, we did not observe clear distinctions in lung function at follow-up when comparing the mild-moderate with the severe ARDS groups.

The carbon monoxide diffusion capacity is a measure of oxygen uptake by the lungs (18), and the test has become helpful in outcome studies of COVID-19 (32). As in our ICU population, DLCO was the test most commonly affected in the ICU cohort in the study by Pini et al. (33). More specifically, in our cohort, patients with more severely widespread opacities had a DLCO reduced to approximately 60%. This relationship was confirmed in regression analyses, where DLCO showed an inverse correlation with residual opacities. In accordance with our findings, Faverio et al. found a similar association and identified mechanical ventilation and older age as risk factors for longstanding pulmonary radiological abnormalities (34). Similarly, in another study of 100 COVID-19 patients with differing disease severities in the acute phase, DLCO correlated inversely with persistent CT ground-glass opacities (35). The authors suggested that the diffuse alveolar damage with loss of alveolar units was the underlying mechanism behind the diffusion impairment.

We show a relatively weak association between PaO_2_/FiO_2_ levels during hospitalisation to DLCO at follow-up. This was confirmed in ROC curves showing a relatively poor association between severe ARDS and reduced DLCO (<60%) or between severe ARDS and high-ROG (>5% opacity) (the accuracy was 64 and 65%, respectively). The strongest association for severe ARDS was observed in relation to severe widespread opacities at follow-up (opacities >25%), with an accuracy of 74%.

In comparison to our study, which focused exclusively on ICU-treated patients, a comprehensive 2 years longitudinal follow-up study in COVID-19 hospitalised patients in Wuhan, China, encompassing various degrees of disease severity, revealed a progressive improvement in functional outcomes over time, irrespective of the severity observed during the acute phase. However, even at the 2 years follow-up mark, up to 50% of patients continued to experience some degree of sequelae, indicative of persistent symptoms associated with long-term COVID-19 (36). These lingering symptoms contributed to a diminished quality of life and limitations in exercise capacity. The authors noted a higher prevalence of lung diffusion impairment in patients who had received ventilatory support for acute respiratory distress syndrome (ARDS) (36). Patients requiring ICU admission and treatment with invasive mechanical ventilation have been shown to exhibit symptoms of post-intensive care syndrome (PICS) during follow-up, which includes cognitive impairment, neuromuscular deconditioning, and psychological disability. Some authors have suggested that COVID-19 critical patients undergoing prolonged mechanical ventilation, sedative use, and steroid treatment may be more susceptible to developing PICS (37).

The occurrence of PE during hospitalisation was related to a reduction in DLCO, and a high D-dimer (>16.4) showed a significant association with a DLCO below 60%. The long-term persistence of this effect remains to be determined. PE in COVID-19 is often small and peripheral (38). We believe that PE in the acute phase could still affect the lumen of lung capillaries at follow-up, potentially by decreasing the effective surface area of the gas exchange membrane.

Longer hospital stays and female sex were significant predictors of lower DLCO at 9 months after discharge. In COVID-19, men are more prone to develop the severe disease (3), requiring hospitalisation to a larger extent. Nevertheless, in this study, women tended to have a lower diffusion capacity at follow-up, as previously observed by Darlington et al. (39).

The 6MWT is a functional performance test previously used in the follow-up of COVID-19 (40, 41). Our results indicate a moderate reduction in distance and oxygen saturation, which could be related to the severity of lung disease.

Regarding the residual symptoms and health-related quality investigated with CAT and EQ5D-5L, no differences were observed between radiological or ARDS severity groups. This result indicates that either only minor differences in symptoms existed or that these questionnaires were not sensitive enough to detect differences. However, and not surprisingly, breathlessness and fatigue at follow-up were associated with more severe acute disease.

## Limitations

There are some limitations that need to be acknowledged in the present study. There might be a selection bias in the recruitment of patients. The study is based on a single centre and might not represent the broader population, especially considering regional differences in COVID-19 treatment and patient demographics. Pulmonary imaging was available in 75% of the patients during hospitalisation. As expected, lung parenchymal opacities were more marked during hospitalisation than at follow-up; however, the time course and evolution could not be predicted. Thereby, the timing of CT and the number of examinations varied considerably, making it difficult to develop a general picture for the cohort. Only one physician assessed the CT scans for graded opacity severity at follow-up, which could affect the reproducibility of our results and introduce subjectivity. Some patients were lost to follow-up during the study period. Some data are missing at the 15 months follow-up. A selection bias might be present at the 2 years follow-up CT. Multiple tests were performed in the analysis and may affect the significance of the results.

## Conclusion and summary

In our cohort, the ARDS severity based on oxygenation criteria did not correspond to major functional impairment at follow-up.

The presence of residual widespread pulmonary radiological abnormalities could clarify functional impairment and persisting symptoms at follow-up. The abnormalities, however, improved over time, and only in a minority of the population did they progress further after 1 year of follow-up.

Residual radiological abnormalities in ICU-treated COVID-19 patients warrant a careful follow-up, regardless of ARDS severity at hospitalisation.

## Data availability statement

The raw data supporting the conclusions of this article will be made available by the authors, upon reasonable request to the corresponding author.

## Ethics statement

The studies involving humans were approved by Swedish ethical review authority, Uppsala, Sweden. The studies were conducted in accordance with the local legislation and institutional requirements. The participants provided their written informed consent to participate in this study. Written informed consent was obtained from the individual(s) for the publication of any potentially identifiable data included in this article.

## Author contributions

MB: Conceptualization, Investigation, Writing – review & editing, Visualization. AS: Conceptualization, Validation, Visualization, Writing – review & editing. CH: Conceptualization, Writing – review & editing, Investigation. MS: Conceptualization, Writing – review & editing, Funding acquisition, Supervision, Validation. SN: Conceptualization, Funding acquisition, Supervision, Validation, Writing – review & editing, Methodology. MN-B: Funding acquisition, Investigation, Methodology, Project administration, Resources, Writing – review & editing, Formal analysis. JB: Conceptualization, Funding acquisition, Methodology, Supervision, Validation, Writing – review & editing, Investigation, Project administration, Resources. MR: Conceptualization, Funding acquisition, Supervision, Validation, Writing – review & editing, Data curation, Formal analysis, Investigation, Project administration, Resources. FJ: Resources, Writing – review & editing, Formal analysis, Investigation, Conceptualization, Validation, Visualization. AK: Conceptualization, Investigation, Writing – review & editing, Data curation, Formal analysis, Funding acquisition, Methodology, Project administration, Resources, Supervision, Validation, Visualization, Writing – original draft.

## Glossary

ARDS: Acute respiratory distress syndrome
CAT: COPD assessment test
CI: Confidence intervals
COPD: Chronic obstructive pulmonary disease
CRP: C-reactive protein
CT: Computed tomography
DLCO: Lung diffusion capacity
ECMO: Extracorporeal membrane oxygenation
EQ-5: Standardised health quality of life questionnaire
ICU: Intensive care unit
IL: Interleukin
IQRs: Interquartile ranges
MIP: Maximal inspiratory pressure
NIV: Non-invasive ventilation
PaO_2_/FiO_2_: Quotient between arterial partial oxygen pressure/oxygen inspired fraction
PE: Pulmonary embolism
ROC: Receiver operating characteristic
ROG: Radiology opacity grade
6MWT: 6 min walk test
