# Supplementary material for: Residual radiological opacities correlate with disease outcomes in ICU-treated COVID-19
Source: Front Med (Lausanne). 2024 Apr 3;11:1263511. doi: 10.3389/fmed.2024.1263511 (PMC11021575; doi:10.3389/fmed.2024.1263511)
Supplement: Supplementary file 1 [file Data_Sheet_1.docx]

**Supplemental table 1 Additional Data from follow–up; demographics, Spirometry outcome**

| **FOLLOW – UP** | ***Full cohort*** | **Low-ROG group** | **High-ROG**  **group** | p–value | **Mild–Moderate ARDS** | **Severe ARDS** | p–value |
| --- | --- | --- | --- | --- | --- | --- | --- |
| **Follow–up 3–month visit Demographics** |  |  |  |  |  |  |  |
| Time after discharge (months) | ´ 2.9 (2.4–3.7) | 3.0 (2.4–4.1) | 2.9 (2.3–3.6) | 0.123 | 2.6 (2.1–3.5) | 2.9 (2.4–3.7) | 0.29 |
| Weight (kg), n=106 | 82 (75–94) | 87 (77–95) | 81 (73–89) | *0.040* | 84 (73–95) | 81 (76–90) | 0.50 |
| Weight change since prehospitalisation (kg) | –6 (–11– (–1)) | –5 (–9– +2) | –7 (–14– (–2) | 0.31 | – 5 (–11–0) | –6 (–10– (–1) | 0.87 |
| BMI (kg/m^2^) | 29 (26–31) | 30 (27–32) | 29 (26–31) | 0.08 | 29 (25–32) | 29 (26–31) | 0.73 |
| Resting SpO_2_ (%) | 98 (97–98) | 98 (97–99) | 98 (97–98) | 0.09 | 97 (97–98) | 98 (97–98) | 0.39 |
| **Follow–up 9 -month visit** |  |  |  |  |  |  |  |
| Time after discharge (months) n=92 | 9 (8–10) | 10 (8–11) | 9 (8–10) | 0.63 | 9 (8–10) | 9 (8–10) | 0.38 |
| Weight (kg) | 93 (79–99) | 97 (88–100) (n=15) | 89 (77–98) (n=28) | 0.19 | 93 (77–99) (n=16) | 93 (80–99) (n=29) | 0.46 |
| BMI (kg/m^2^) | 31 (28–33) | 32 (30–33) | 28 (26–31) | 0.35 | 30 (28–33) | 31 (29–33) | 0.34 |
| Weight change since prehospitalisation (kg) | 0 (–5–6) | 0 (–6–6) | 1 (–2–7) | 0.94 | 4 (3–6) | 7 (3–13) | 0.25 |
| Weight change since between 3- and 9~~-~~month visit (kg) | 5 (3–12) | 5 (3–6) | 7 (3–11) | 0.77 | –2 (–8–4) | 0.6 (–2–8) | 0.065 |
|  |  |  |  |  |  |  |  |
| **SPIROMETRY Dynamic**, **9-month visit** n=110 |  |  |  |  |  |  |  |
| Vital capacity (L) % of predicted | 83 (79–86) | 88 (82–93) | 81 (76–85) | *0.047* | 83 (78–88) | 83 (78–87) | 0.97 |
| Residual volume (L) % of predicted | 87 (71–104) | 113 (95–131) | 82 (66–99) | *<0.001* | 99 (80–118) | 89 (82–97) | 0.23 |
| Max inspiratory pressure, n=47 | 99 (88–110) | 105 (87–123) | 96 (82–110) | 0.36 | 93 (677–110) | 102 (87–117) | 0.93 |
|  |  |  |  |  |  |  |  |
| **Dynamic,** **15–month visit** n=51, |  |  |  |  |  |  |  |
| Vital capacity (L) % of predicted | 82 (78–87) | 89 (80–99) | 80 (75–85) | 0.08 | 82 (74–90) | 82 (77–87) | 0.96 |
| Residual volume (L) % of predicted | 102 (90–114) | 127 (88–166) | 95 (84–106) | *<0.05* | 107 (69–145) | 101 (88–113) | 0.66 |

ARDS= acute respiratory distress syndrome, High-ROG= Patients with more widespread opacity changes on 10-month CT; Low-ROG=patients with no/minor opacities in 10 months CT; ‡ max inspiratory pressure and max expiratory pressure presented according to actual values

**Supplemental table 2 Data from follow–up; 6- min Walk test and CAT additional outcome data**

| **FOLLOW – UP** | ***Full cohort*** | **Low-ROG group** | **High-ROG**  **group** | p–value | **Mild–Moderate ARDS** | **Severe ARDS** | p–value |
| --- | --- | --- | --- | --- | --- | --- | --- |
| **FOLLOW – UP 6 min Walk test** |  |  |  |  |  |  |  |
| **3-month follow-up** |  |  |  |  |  |  |  |
| Time after discharge (days) | ´ 2.9 (2.4–3.7) | 3.0 (2.4–4.1) | 2.9 (2.3–3.6) | 0.12 | 2.6 (2.1–3.5) | 2.9 (2.4–3.7) | 0.37 |
| Heart rate at 1 min (beats/min) | 98 (94–101) | 101 (94–107) | 97 (92–102) | 0.35 | 95 (87–104) | 99 (86–113) | 0.27 |
| Heart rate at 2 min (beats/min) | 100 (95–105) | 104 (84–116) | 100 (95–106) | 0.86 | 96 (85–106) | 101 (88–116) | 0.24 |
| SpO_2_ at 2 min (%) | 94 (93–95) | 99 (90–97) | 94 (93–95) | 0.09 | 95 (94–96) | 94 (93–95) | 0. 38 |
| Heart rate at 3 min (beats/min) | 102 (97–107) | 101 (92–110) | 102 (96–108) | 0.87 | 95 (84–106) | 107 (91–119) | 0.08 |
| SpO_2_ at 3 min (%) | 94 (93–95) | 96 (95–97) | 93 (92–94) | *<0.001* | 95 (93–97) | 94 (93–95) | 0.17 |
| Heart rate at 4 min (beats/min) | 102 (97–107) | 104 (96–114) | 101 (95–107) | 0.53 | 98 (87–109) | 107 (87–117) | 0.26 |
| SpO_2_ at 4 min (%) | 95 (93–97) | 96 (96–97) | 94 (93–95) | *<0.01* | 96 (95–97) | 94 (93–95) | 0.07 |
| Heart rate at 5 min (beats/min) | 103 (98–108) | 105 (87–123) | 102 (95–108) | 0.54 | 98 (86–122) | 107 (90–119) | 0.22 |
| SpO_2_ at 5 min (%) | 94 (93–95) | 96 (96–97) | 93 (92–94) | *<0.001* | 95 (94–97) | 94 (93–95) | 0.22 |
| Heart rate at 6 min (beats/min) | 107 (102–111) | 110 (102–118) | 105 (100–111) | 0.30 | 102 (93–112) | 111 (97–123) | 0.21 |
| Highest SpO_2_ (%) | 96 (96–97) | 98 (97–98) | 95 (95–96) | *<0.001* | 96 (96–97) | 96 (95–97) | 0.35 |
| Lowest SpO_2_ (%) | 94 (94–95) | 94 (93–96) | 92 (91–93) | *<0.01* | 93 (92–94) | 92 (91–93) | 0.41 |
| Mean SpO_2–_ drop (levels) | 4 (3–4) | 2 (1–3) | 4 (3–5) | 0.29 | 3 (2–5) | 4 (3–5) | 0.70 |
| **9-month follow-up** |  |  |  |  |  |  |  |
| SpO_2_ at 2 min (%) | 95 (91–100) | 99 (98–100) | 97 (95–98) | 0.22 | 98 (97–100) | 94 (87–100) | 0.27 |
| SpO_2_ at 3 min (%) | 97 (96–98) | 99 (98–100) | 97 (95–98) | *0.01* | 99 (98–100) | 97 (95–98) | 0.12 |
| SpO_2_ at 4 min (%) | 97 (96–98) | 99 (98–100) | 96 (95–98) | *0.03* | 98 (96–100) | 96 (95–98) | 0.33 |
| SpO_2_ at 5 min (%) | 97 (96–98) | 99 (98–100) | 96 (95–98) | *0.02* | 99 (97–100) | 96 (95–98) | 0.44 |
| Time after discharge (days) | 10 (9–12) | 11 (10–12) | 10 (9–12) | 0.63 | 10.5 (9–12) | 10 (9–12) | 0.38 |
| Highest SpO_2_ (%) | 99 (98–99) | 100 (99–100) | 98 (98–99) | *0.05* | 99 (99–100) | 98 (98–99) | 0.35 |
| Lowest SpO_2_ (%) | 94 (90–99) | 98 (96–99) | 92 (85–99) | 0.21 | 98 (96–99) | 92 (86–99) | 0.06 |
|  |  |  |  |  |  |  |  |
| **BORG scale (CR–10 and RPE–scale) during 6 MWT, 3–month visit** |  |  |  |  |  |  |  |
| Resting Breathlessness | 0 (0–2) | 0 (0–1) | 0 (0–2) | 0.43 †† | 0 (0–2) | 0 (0–2) | 0.70 †† |
| Resting fatigue | 0 (0–2) | 0 (0–1) | 0 (0–2) | 0.73 †† | 0 (0–1) | 0 (0–2) | 0.30 †† |
| Resting chest tightness | 6 (6–9) | 6 (6–7) | 6 (6–9) | 0.36 †† | 6 (6–10) | 6 (6–9) | 0.55 †† |
|  |  |  |  |  |  |  |  |
| Breathlessness during 6 MWT | 3 (3–5) | 3 (2–5) | 4 (3–5) | 0.34 †† | 3 (3–5) | 4 (3–5) | 0.88 †† |
| Fatigue during 6 MWT | 3 (2–5) | 3 (2–6) | 3 (2–4.5) | 0.49 †† | 3 (1.5–6) | 3 (2–5) | 0.73 †† |
| Chest tightness during 6 MWT | 13 (11–15) | 13 (11–15) | 13 (11–15) | 0.70 †† | 13 (11–15) | 13 (11–15) | 0.27 †† |
| **CAT at Follow–up** | ***Full cohort***  ***Breathlessness*** | ***Full cohort***  ***Fatigue*** | ***Full cohort***  ***Cough*** | ***Full cohort***  ***Chest tightness*** |  |  |  |
| **3–month visit, (n= 106) stating** |  |  |  |  |  |  |  |
| no 0, nr (%) | 5 (5) | 8 (8) | 18 (17) | 37 (35) |  |  |  |
| 1 | 13 (12) | 11 (10) | 29 (27) | 19 (18) |  |  |  |
| 2 | 14 (13) | 23 (22) | 18 (17) | 17 (16) |  |  |  |
| 3 | 27 (25) | 36 (34) | 15 (14) | 11 (10) |  |  |  |
| 4 | 25 (24) | 22 (21) | 7 (7) | 3 (3) |  |  |  |
| 5 | 22 (21) | 5 (5) | 3 (3) | 2 (2) |  |  |  |
|  |  |  |  |  |  |  |  |
| **9-month visit (n=90)** |  |  |  |  |  |  |  |
| no 0, nr (%) | 8 (11) | 7 (8) | 19 (21) | 30 (33) |  |  |  |
| 1 | 20 (22) | 18 (20) | 28 (31) | 20 (22) |  |  |  |
| 2 | 17 (19) | 21 (23) | 20 (27) | 16 (18) |  |  |  |
| 3 | 23 (26) | 18 (20) | 6 (7) | 6 (7) |  |  |  |
| 4 | 16 (18) | 10 (11) | 2 (2) | 3 (4) |  |  |  |
| 5 | 6 (7) | 4 (4) | 3 (3) | 1 (1) |  |  |  |

Demographics are expressed as median (interquartile range). ARDS= acute respiratory distress syndrome, BMI=body mass index. High-ROG= Patients with more widespread opacity changes on 10-month CT; Low-ROG=patients with no/minor opacities in 10 months CT

CR–10; Borg CR-10 scale; RPE–scale; Rated perceived exertion scale;

The figures represent % (nr) of patients stating the symptoms breathlessness and fatigue, cough, and chest tightness in grades ranging from 0 (none) to 5 (severe). For severe breathlessness and severe fatigue expressed as % affected, n= affected subjects in each group/ not affected subjects.

Breathlessness grading according to grade: 0= “I have no breathlessness when walking in stairs or up a hill”, grade 5= “When walking in stairs or up a hill I get severe breathlessness”.

Fatigue grading according to grading; grade: 0= “I have lots of energy”, grade 5= “I do not have any energy”.

Cough grading according to grading; grade: 0= “I never cough” grade 5= “I cough constantly”.

Chest tightness grading according to grading; grade: 0=” I feel no chest tightness at all” grade 5=” I fell a lot of chest tightness”.

ARDS= acute respiratory distress syndrome, CAT=COPD assessment test; High-ROG= Patients with more widespread opacity changes on 10-month CT; Low-ROG=patients with no/minor opacities in 10 months CT

Scores presented as median (IQR). P <0.01 was considered significant. Statistical analyses are based on † Pearson chi–squared for categorical data. †† Mann Whitley U test for non–parametric values.

**Supplemental Table S3.** Patients treated on ECMO during hospitalization **c**ompared with the rest of the cohort.

|  | **ECMO**  **(n=8)** | **Non–ECMO**  **(n=110)** | **p–value** |
| --- | --- | --- | --- |
| Age | 47 (44–52) | 59 (51–65) | *<0.01* |
| D–dimer (mg/L) | 23 (11–35) | 6.7 (3.7–13.7) | *<0.01* |
| Time on mechanical ventilation (days) | 44 (41–55) | 12 (8–22) | *<0.001* |
| ICU LOS (days) | 46 (45–56) | 15 (10–25) | *<0.001* |
| Time in ECMO (days) | 22 (10–35) | 0 | – |
| Nr classified as high-ROG group, % yes (nr yes/no) | 8/8 | 73/110 | *0.044 †* |
| Predicted DLCO 9 -month visit (n=8) | 58 (49–66) | 81 (69–90) | *<0.001* |
| Predicted DLCO 15- month visit (n=5) | 62 (53–68) | 80 (73–89) | *<0.01* |
| 6 MWT Distance (meters) 3–month visit (n=8) | 303 (250– 420) | 480 (380–550) | *0.013* |
| Distance of ref (%) * | 48 (39–87) | 85 (71–97) | 0.33 |

DLCO= diffusion capacity; ECMO= Extracorporeal membrane oxygenation; ICU=intensive care unit; LOS= Length of hospital stay; 6 MWT= 6-minute walk test * According to formula see material and methods.

P <0.05 was considered significant. Statistical analyses are based on analysis of variances; Student t–test † Pearson chi–squared for categorical data.

**Supplemental Table S4.** Odds Ratio analysis with DLCO at 9–month visit as dependent variable.

| **n=108** | **n** | **Above** | **Odds Ratio** | Upper CL 95% | Lower CL 95% | p |
| --- | --- | --- | --- | --- | --- | --- |
| **Variable** |  |  |  |  |  |  |
| Total hospital stay (in days) | 108 | >57 days | 6.00 | 1.198 | 30.08 | 0.029 |
| ICU time | 108 | >42 days | 2.29 | 0.396 | 13.19 | 0.36 |
| PaO_2_/FiO_2_ | 108 | <8.1 | 0.96 | 0.209 | 4.437 | 0.96 |
| D-dimer | 108 | >17.5 | 1.73 | 0.372 | 8.017 | 0.49 |
| CRP | 108 | >408 ug/L | 0.78 | 0.167 | 3.609 | 0.75 |
| Smoking | 108 | Yes/no | 0.74 | 0.208 | 2.643 | 0.65 |
| Female gender | 108 | Women/men | 0.23 | 0.057 | 0.932 | *0.039* |
| Age | 108 | >62 years | 1.01 | 0.268 | 3.794 | 0.99 |

PaO_2_/FiO_2_=quotient between arterial partial oxygen pressure and fraction of inspired oxygen; ICU= intensive care unit, CRP= C reactive protein
