# Supplementary figures and images for: Residual radiological opacities correlate with disease outcomes in ICU-treated COVID-19
Source: Front Med (Lausanne). 2024 Apr 3;11:1263511. doi: 10.3389/fmed.2024.1263511 (PMC11021575; doi:10.3389/fmed.2024.1263511)

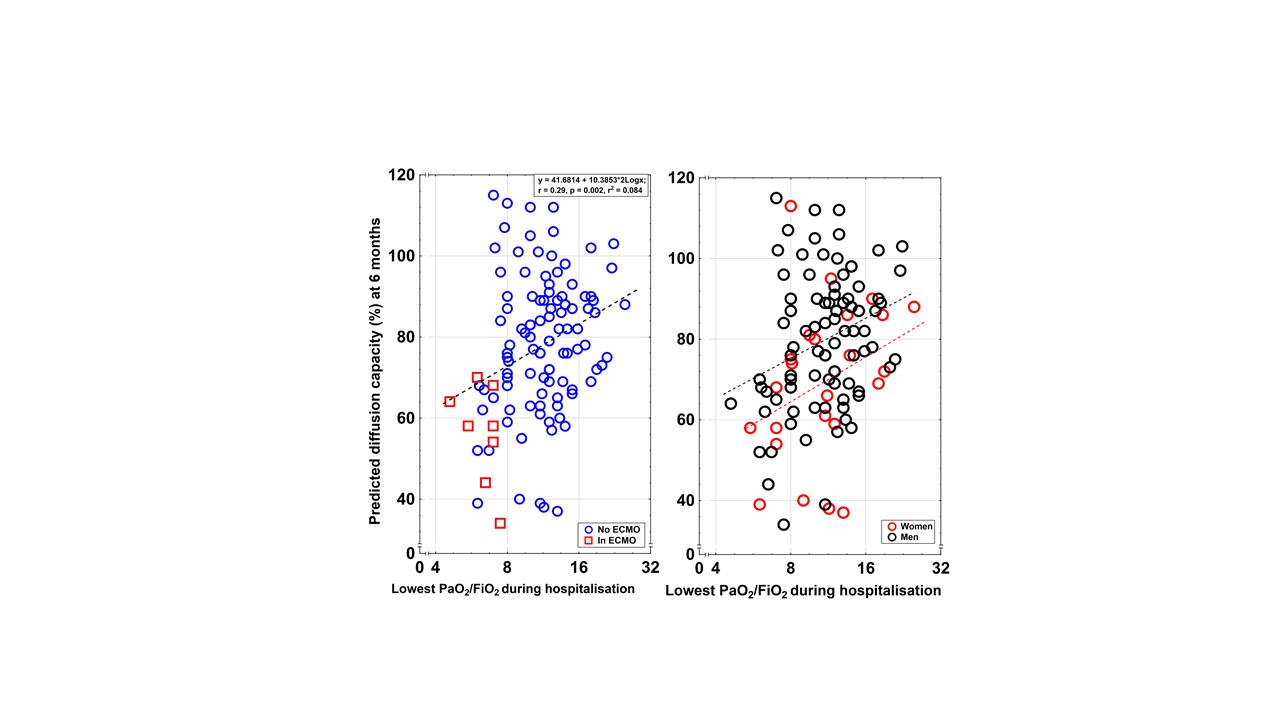

Supplement: Supplementary file 2 [file Image_1.JPEG]
